# Acute Deep Vein Thrombosis Presents as an Early Complication of Wilson Disease

**DOI:** 10.7759/cureus.83037

**Published:** 2025-04-26

**Authors:** Aseel Faroun, Roua Faroun, Rania Mashal, Mutaz Sultan

**Affiliations:** 1 Medicine, Al-Quds University, Jerusalem, PSE; 2 Pediatric Gastroenterology, Al-Makassed Hospital, Al-Quds University, Jerusalem, PSE

**Keywords:** deep vein thrombosis, hepatic coagulopathy, pediatric thrombosis, rare presentation, wilson disease

## Abstract

Wilson disease (WD) is a rare, autosomal recessive disorder of copper metabolism. Although hepatic and neuropsychiatric manifestations are common, thrombotic events such as deep vein thrombosis (DVT) are exceedingly rare, particularly in pediatric patients.

We present the case of an 11-year-old girl diagnosed with WD who developed an acute left upper limb DVT shortly two months after diagnosis. Laboratory and imaging findings confirmed hepatic involvement and coagulopathy. Genetic testing revealed a heterozygous Factor V Leiden mutation and MTHFR heterozygosity; both may have contributed to the thrombotic episode.

Careful monitoring of WD was prioritized, and appropriate treatment plans were established during diagnosis to achieve satisfactory outcomes and prevent future thrombotic sequences. This case emphasizes the importance of early recognition of thrombotic complications and coagulopathy management in pediatric WD.

## Introduction

Wilson disease (WD), also known as hepatolenticular degeneration, is a rare autosomal recessive disorder with an estimated prevalence of one in 30,000 individuals worldwide [1]. It is caused by mutations in the ATP7 B gene on chromosome 13, which impairs copper transport and leads to toxic accumulation in organs such as the liver, brain, and corneas [1-3]. Patients typically present with hepatic symptoms (e.g., jaundice, ascites, and pruritus), neuropsychiatric features (e.g., tremor and behavioral changes), and ocular findings like Kayser-Fleischer rings [4]. While the age of onset varies widely, most cases are diagnosed between childhood and early adulthood, affecting males and females equally [5]. The clinical presentation often correlates with organ-specific copper deposition. Neurological symptoms are more common in males, whereas hepatic manifestations are more frequent in females [6]. Importantly, hepatic dysfunction may lead to secondary coagulopathy due to impaired synthesis of clotting factors.

However, thrombotic complications such as deep vein thrombosis (DVT) are rarely reported, especially in pediatric cases [7-9]. These events may be linked to underlying liver dysfunction, systemic inflammation, or associated prothrombotic mutations. Our case report aims to highlight an unusual early complication of WD in a child, underscoring the importance of recognizing thrombotic risks even in non-cirrhotic or early-stage patients.

## Case presentation

An 11-year-old Middle Eastern female with WD, diagnosed two months prior, presented with acute left arm DVT. Symptoms began five days before hospital admission, including pain, swelling, warmth, and visible veins in the arm. The patient had no history of trauma, prolonged immobilization, or family history of WD or thrombotic events. She had been compliant with a high penicillamine dose (250 mg * 4) and a low-copper diet since her diagnosis. Upon examination, the patient exhibited yellowish skin, a petechial rash on her ankles, and bilateral lower limb pitting edema (+3). Her abdomen was distended (circumference=70 cm) with dull percussion, but no shifting dullness was noted. Neurological and other system examinations were unremarkable. The patient remained hemodynamically stable with normal vital signs (heart rate: 111 bpm; blood pressure: 120/67 mmHg).

Initial laboratory findings (Table 1) revealed markedly decreased ceruloplasmin (0.03 mg/dL), elevated 24-hour urinary copper excretion (200 mcg/day at baseline; 340 mcg/day post-penicillamine), and elevated liver enzymes (AST 162.8 IU/L, ALT 56 IU/L). Coagulation studies showed prolonged PT (30.6 sec), INR (2.44), and aPTT (47.4 sec), consistent with coagulopathy. Additional abnormalities included hemolytic anemia, thrombocytopenia, elevated D-dimer (681 ng/mL), hypoalbuminemia (2.7 g/dL), and hyponatremia (129 mmol/L). A viral hepatitis panel was negative (Table 2), effectively ruling out infectious causes of acute liver failure. According to the hospital physicians, the patient's clinical picture, including the pitting edema, localized tenderness, arm swelling, and lab results, can establish a +3 score in the modified Wells criteria for DVT [10,11]. Furthermore, a Doppler ultrasonography report recently demonstrated an acute venous thrombosis in the patient's left arm.

**Table 1:** Initial lab test results, which show elevated liver enzymes, hypoalbuminemia, coagulopathy, thrombocytopenia, anemia, and hyponatremia The values in bold in the tables reflect the positive lab results. AST: aspartate aminotransferase; ALT: alanine aminotransferase; PT: prothrombin time; INR: international normalized ratio; aPTT: activated partial thromboplastin time; Hgb: hemoglobin; TIBC: total iron binding capacity

Test name	Normal range (children)	Results
Bilirubin (direct)	0.0-0.3 mg/dL	3.36 mg/dL
Bilirubin (total)	0.3-1.2 mg/dL	4.94 mg/dL
AST	10-40 U/L	162.8 U/L
ALT	10-40 U/L	56 U/L
Albumin	3.5-5.0 g/dL	2.7 g/dL
Total Protein	6-8.3 g/dL	6 g/dL
PT	11-15 sec	30.6 sec
INR	0.9-1.1	2.44
aPTT	24-40 sec	47.4 sec
D-Dimer	<250 ng/dL	681 ng/dL
WBC	4.5-11*10^^9^/L	2.99*10^^9^/L
Neutrophils	2.5-7.0/L	0.877/L
Lymphocytes	3.0-9.5 /L	1.873/l
Platelets	150-450*10^^9^/L	123*10^^9^/L
Hgb	12.1-15.1 g/dL	9.53 g/dL
Reticulocytes %	0.5%-2.5%	3.2 %
Ferritin	13-150 ng/mL	p=0.008
TIBC	50-170 mcg/dL	148 mcg/dL
Blood film		Slight anisocytosis, few ovalocytes and target cells, and rare acanthocytes
Na	135-145 mEq/L	129 mEq/L

**Table 2:** Viral hepatitis panel HAV: hepatitis A virus

EBV IgG	46 (Negative)
EBV IgM	0.10 (Negative)
EBNA-1 IgG	26.28; cut off point is <25 U/mL
HAV IgM	Negative
HAV IgA	Negative
HBsAg	Non-reactive
CMV IgM	0.14 (Negative)
CMV IgG	111 (then avidity test was ordered and results were negative)

**Figure 1:**
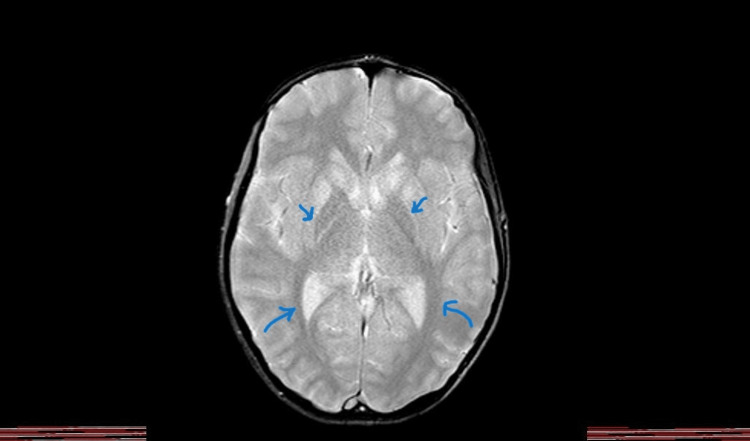
Brain MRI shows symmetric T2 and FLAIR hyperintense signals in the caudate nucleus and putamen

Imaging included brain MRI, which showed symmetric T2 and FLAIR hyperintense signals in the caudate nucleus and putamen, suggesting central nervous system (CNS) involvement by WD. There was no midline shift or abnormal findings in the paranasal sinuses or mastoid air cells, as shown in Figure 1.

During the patient's hospitalization, management involved controlling symptoms, close monitoring of liver function, and electrolyte status. Given the context of acute liver failure and a confirmed diagnosis of DVT, anticoagulation therapy was initiated. Despite the inherent bleeding risk associated with hepatic dysfunction, the elevated D-dimer and confirmed DVT warranted anticoagulation, which was commenced following multidisciplinary team consultation. The therapeutic regimen included zinc sulfate, penicillamine, and ursodeoxycholic acid (Ursolit), alongside supportive care with hypertonic saline to correct hyponatremia. The patient responded well to treatment, with notable improvement in clinical symptoms and laboratory parameters.

By discharge, DVT symptoms had resolved, and liver function markers had stabilized. She was prescribed a comprehensive regimen, including vitamin K, zinc sulfate, penicillamine, ursodeoxycholic acid, and anticoagulants. On follow-up, the patient remained clinically stable, with complete resolution of arm swelling and pain. Genetic testing confirmed the diagnosis of WD (Table 3) and identified a heterozygous Factor V Leiden mutation (Table 4), supporting a prothrombotic state. The presence of both hepatic coagulopathy and inherited thrombophilia likely contributed synergistically to the early onset of DVT in this pediatric patient.

**Table 3:** Details of SNV results SNP: single nucleotide polymorphism; dbSNP: single nucleotide polymorphism database; ACMG: American College of Medical Genetics and Genomics

Gene	Transcript	Position	Nucleotide	dbSNP	Zygosity	ACMG classification
ATP7B	NM_0000S3.4	13:52534477	c.1947-19T>A	Rs1593733949	HOM	Likely Pathogenic

**Table 4:** Thrombophilia panel for CVD ^*^Test methodology The test was performed using a real-time PCR machine according to the manufacturer's instructions, with the Thrombophilia Multiplex Real-Time PCR Kit B (Cat. No: 10R-20-12B) from SNP Biotechnology. MTRR: methionine synthase reductase; MTR: methionine synthase (5-methyltetrahydrofolate-homocysteine methyltransferase)

Genetic factor*	Results
FII Prothrombin (Ala20210Gly)	Normal
MTHFR (Ala1298Cys)	Heterozygous
FXIII (Val34Leu)	Heterozygous
FV (Tyr1702Cys)	Normal
Factor V Leiden (FV Arg506Gln)	Heterozygous
FV (His1299Arg)	Normal
Factor V Cambridge (FV Arg306Thr)	Normal
MTR (Ala2756…	Normal
MTHFR (C677T)	Normal
PAI-1 5G/4G	Homozygous
B-Fibrinogen (Gly544Ala)	Heterozygous
MTRR (Ala66Gly)	Normal

## Discussion

We describe a rare case of an 11-year-old female with WD presenting with acute DVT of the left upper limb. Her initial symptoms included pain, swelling, and warmth in the left arm, along with jaundice, ascites, and bilateral lower limb pitting edema. WD had been diagnosed two months prior, and subsequent workup revealed acute liver failure, hemolytic anemia, thrombocytopenia, and coagulopathy. Viral hepatitis was excluded through a negative serologic panel [10-13].

WD arises from mutations in the ATP7 B gene, which impair biliary copper excretion and lead to toxic copper accumulation. Copper-induced oxidative stress damages hepatocytes, potentially leading to a clinical spectrum ranging from acute liver failure to cirrhosis or neuropsychiatric manifestations [1,14]. The elevated INR and prolonged PT observed, in this case, reflect impaired hepatic synthetic function, while concurrent thrombocytopenia and anemia further support evolving liver failure. Hyponatremia was likely secondary to both high-dose penicillamine and hypoalbuminemia-induced third spacing, which are commonly seen in decompensated hepatic states.

While liver disease is typically associated with bleeding risks, paradoxically, it also predisposes patients to thrombosis due to reduced synthesis of anticoagulant proteins such as protein C, protein S, and antithrombin III [15]. This complex coagulopathy poses significant challenges when initiating anticoagulation therapy. In this case, the decision to start anticoagulation was carefully weighed against the bleeding risk and was ultimately guided by the confirmed presence of DVT and the need to prevent further thrombotic events. Although the patient did not exhibit advanced hepatic failure, the underlying coagulopathy warranted anticoagulation, which was closely monitored during treatment.

Additional workup identified multiple genetic thrombophilias that likely contributed to the thrombotic event. The patient was heterozygous for the Factor V Leiden (Arg506Gln) mutation and MTHFR A1298C variant and homozygous for the PAI-1 4G/5G polymorphism. Each of these mutations is known to increase thrombotic risk, and their presence in a patient with WD-associated coagulopathy underscores the multifactorial nature of her presentation [16,17]. These findings emphasize the importance of comprehensive thrombophilia screening in pediatric patients with WD and unexplained thrombotic complications [18].

Although uncommon, cases of DVT in WD have been reported. One such instance involved a 32-year-old male who presented with right-leg DVT as the initial manifestation of WD [19]. He exhibited prolonged PT, thrombocytopenia, and elevated factor VIII and von Willebrand factor levels, findings consistent with copper-induced hepatic injury and coagulopathy. The rarity of such cases reinforces the importance of maintaining a high index of suspicion for thrombotic events in patients with early-stage WD.

Management of coagulopathy in pediatric WD requires individualized approaches, with careful monitoring of coagulation parameters and judicious use of vitamin K to support hepatic synthesis of clotting factors. Anticoagulants, such as low-molecular-weight heparin or warfarin, may be used cautiously but require close surveillance due to the bleeding risk associated with liver dysfunction [19]. Current guidelines recommend tailoring treatment based on the severity of hepatic impairment, degree of coagulopathy, and the patient’s thrombotic risk profile [20].

## Conclusions

This case highlights an unusual presentation of pediatric WD with early-onset DVT preceding advanced hepatic decompensation. The presence of inherited thrombophilia, including heterozygous Factor V Leiden and the MTHFR A1298C mutation, may have contributed to the patient’s thrombotic risk in the setting of liver dysfunction. Clinicians should be aware of the potential for thrombotic complications, even in early-stage WD, and consider routine coagulation screening and thrombophilia evaluation. Early intervention with vitamin K, anticoagulation, and tailored copper chelation therapy contributed to favorable clinical outcomes in our case.
